# Preparation of Pendant Group-Functionalized Diblock Copolymers with Adjustable Thermogelling Behavior

**DOI:** 10.3390/polym9060239

**Published:** 2017-06-20

**Authors:** Bo Keun Lee, Ji Hoon Park, Seung Hun Park, Jae Ho Kim, Se Heang Oh, Sang Jin Lee, Bun Yeoul Lee, Moon Suk Kim

**Affiliations:** 1 Department of Molecular Science and Technology, Ajou University, Suwon 443-749, Korea; acousticjazz@ajou.ac.kr (B.K.L.); jhp@ajou.ac.kr (J.H.P.); hpt88@ajou.ac.kr (S.H.P.); jhkim@ajou.ac.kr (J.H.K.); bunyeoul@ajou.ac.kr (B.Y.L.); 2 Department of Nanobiomedical Science, Dankook University, Cheonan 330-714, Korea; seheangoh@dankook.ac.kr; 3 Wake Forest Institute for Regenerative Medicine, Winston-Salem, NC 27157, USA; sjlee@wfubmc.edu

**Keywords:** thermogelling, diblock copolymer, pendant group, crystallinity, phase transition

## Abstract

Recently, several thermogelling materials have been developed for biomedical applications. In this study, we prepared methoxy polyethylene glycol (MPEG)-*b*-(poly(ε-caprolactone)-*ran*-poly(2-chloride-ε-caprolactone) (PCL-*ran*-PfCL)) (MP-Cl) diblock copolymers at room temperature via the ring-opening polymerization of caprolactone (CL) and 2-chloride-ε-caprolactone (fCL) monomers, using the terminal alcohol of MPEG as the initiator in the presence of HCl. MPEG-*b*-(poly(ε-caprolactone)-*ran*-poly(2-azide-ε-caprolactone) (PCL-*ran*-PCL-N_3_)) (MP-N_3_) was prepared by the reaction of MP-Cl with sodium azide. MPEG-*b*-(poly(ε-caprolactone)-*ran*-poly(2-amine-ε-caprolactone) (PCL-*ran*-PCL-NH_2_)) (MP-NH_2_) was subsequently prepared by Staudinger reaction. MP-Cl and MP-N_3_ showed negative zeta potentials, but MP-NH_2_ had a positive zeta potential. MP-Cl, MP-N_3_, and MP-NH_2_ solutions formed opaque emulsions at room temperature. The solutions exhibited a solution-to-hydrogel phase transition as a function of the temperature and were affected by variation of the chloride, azide, and the amine pendant group, as well as the amount of pendant groups present in their structure. Additionally, the phase transition of MP-Cl, MP-N_3_, and MP-NH_2_ copolymers was altered by pendant groups. The solution-to-hydrogel phase transition was adjusted by tailoring the crystallinity and hydrophobicity of the copolymers in aqueous solutions. Collectively, MP-Cl, MP-N_3_, and MP-NH_2_ with various pendant-group contents in the PCL segment showed a solution-to-hydrogel phase transition that depended on both the type of pendant groups and their content.

## 1. Introduction

Over the past few decades, several thermogelling materials have been investigated extensively for a broad range of biomedical applications, such as in drug- or cell-delivery carriers [1]. Thermogelling materials can absorb water and swell in aqueous environments, which allow them to retain large amounts of fluids [2,3,4]. Fully hydrated thermogelling material solutions can incorporate various therapeutic agents such as drugs, growth factors, gene products, or cells by simple mixing. Thermogelling material solutions can be injected and solidified at the specific injected body site [5,6,7].

Various thermogelling materials have been developed. Among them, block copolymers consisting of (methoxy)polyethylene glycol (MPEG), poly(propylene oxide), and biodegradable polyesters, such as poly(l-lactic acid) (PLLA), poly(glycolic acid), their copolyesters, or poly(ε-caprolactone) (PCL), have been reported as potential thermogelling candidates [8,9,10,11,12].

Previously, we developed MPEG-*b*-PCL (MP) and MPEG-*b*-polyester diblock copolymers, (MPEG-*b*-(PCL-*ran*-PLLA), MPEG-*b*-(PCL-*ran*-poly(trimethylene carbonate)), and MPEG-*b*-(PCL-*ran*-poly(1,4-dioxan-2-one)), as thermogelling materials [13,14,15,16].

Among these, the MP material exhibited favorable thermogelling properties, such as long-term gel persistence. Furthermore, MP was synthesized by ring-opening polymerization of ε-caprolactone (CL) in the presence of HCl·Et_2_O as monomer activator [13]. This ring-opening polymerization is especially attractive for biomedical application because HCl·Et_2_O can be easily removed from MP.

Recently, we examined the thermogelling properties of MP diblock copolymers derivatized with a carboxylic acid group, an amine group, and a zwitterionic group at the end of an MP chain [17,18]. The functional groups on MP diblock copolymers engaged in intra- and inter-molecular interactions to stabilize or destabilize diblock copolymer chain aggregates. The end-group derivatization of MP diblock copolymers can lead to macroscopic gelation through the formation of intra- and inter-molecular interactions.

In a later study, we prepared thermogelling diblock copolymers using benzyl, hydroxyl or carboxylic acid groups in the pendant-position derivatization of diblock copolymers [19]. We found that intra- and inter-molecular interactions among hydrophobic PCL segments can stabilize or destabilize aggregation depending on functional pendant groups.

Based on these results, we concluded that the formation or destruction of a structured network of PCL hydrophobic blocks depends on the functional groups in the chain end or the pendant positions and thus induces different thermal phase-transition behaviors.

**Figure 1:**
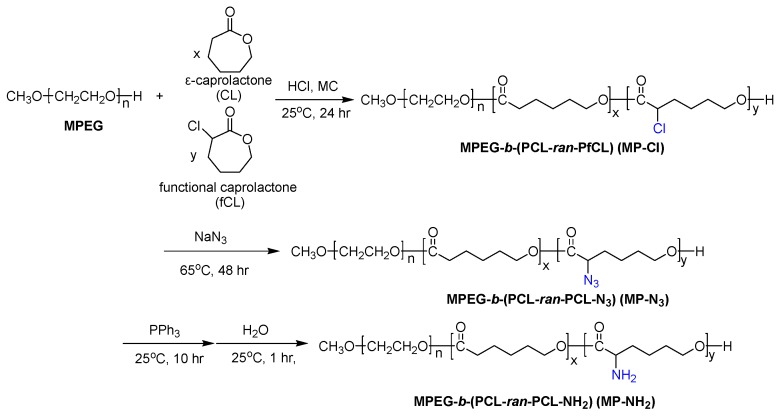
Synthesis of MP-Cl, MP-N_3_ and MP-NH_2_ diblock copolymers.

In this work, on the basis of previous studies, we introduced different pendant groups into MP to alter the thermogelling behavior of the MP copolymer in aqueous solutions, as shown in Figure 1. We firstly describe the ring-opening polymerization of CL and 2-chloride-ε-caprolactone (fCL) monomers by metal-free method in the presence of HCl·Et_2_O. The ROP of CL and fCL were randomly copolymerized with varying feed ratios to prepare MPEG-*b*-(poly(ε-caprolactone)-*ran*-poly(2-chloride-ε-caprolactone)) (MPEG-*b*-(PCL-*ran*-PfCL)) (MP-Cl) diblock copolymers.

Next, MPEG-*b*-(poly(ε-caprolactone)-*ran*-poly(2-azide-ε-caprolactone)) (MPEG-*b*-(PCL-*ran*-PCL-N_3_)) (MP-N_3_) was prepared by the reaction of MP-Cl with sodium azide. Subsequent Staudinger reaction yielded MPEG-*b*-(poly(ε-caprolactone)-*ran*-poly(2-amine-ε-caprolactone)) (MPEG-*b*-(PCL-*ran*-PCL-NH_2_)) (MP-NH_2_). The effects of chloride, azide and amine pendant groups on intra- and inter-molecular interactions were tested. Then, we examined the solution-to-hydrogel phase transitions of MP-Cl, MP-N_3_, and MP-NH_2_ as a function of temperature to understand how the type and amount of pendant groups and the solubility and crystallinity of MP-Cl, MP-N_3_, and MP-NH_2_ stabilize or destabilize aggregation.

## 2. Materials and Methods

### 2.1. Materials

MPEG (number-average molecular weight *M*_n_ = 750, Sigma, St. Louis, MO, USA) and HCl (Aldrich; 1.0 M solution in diethyl ether, Sigma, St. Louis, MO, USA) were used as received. ε-Caprolactone (CL) (TCI, Tokyo, Japan) was distilled over CaH_2_ at a reduced pressure. 2-Chlorocyclohexanone (TCI, Tokyo, Japan), 3-chloroperoxybenzoic acid (Sigma, St. Louis, MO, USA), sodium azide (Sigma, St. Louis, MO, USA), and triphenylphosphine (Sigma, St. Louis, MO, USA) were used as received. CH_2_Cl_2_ was distilled sequentially from CaCl_2_ and CaH_2_ in a nitrogen atmosphere before use.

### 2.2. Characterization

^1^H-NMR and ^13^C-NMR spectra were measured using a Varian Mercury Plus 400 (Oxford Instruments, Abingdon, UK) with CDCl_3_ in the presence of Tetramethylsilian (TMS) as an internal standard. The molecular-weight distributions of MP-Cl, MP-N_3_, and MP-NH_2_ were measured at 40 °C using a YL-Clarity GPC system (YL 9170 RI detector) equipped with three columns (Shodex K-802, K-803 and K-804 polystyrene gel columns). For this measurement, CHCl_3_ was used as the eluent, at a flow rate of 1.0 mL/min, and polystyrene was used for calibration. FT-IR spectra were measured by using a Thermo Nicolet 6700 spectrometer (Thermo Electron Corporation, Runcorn, UK). The melting temperature (*T*_m_) and the heat of fusion (Δ*H*_m_) of MP-Cl, MP-N_3_ and MP-NH_2_ were determined using differential scanning calorimetry (DSC; Q1000, TA Instruments, Eschborn, Germany) performed from −80 to 200 °C at a heating rate of 5 °C/min in a nitrogen atmosphere. DSC of MP-Cl, MP-N_3_, and MP-NH_2_ in aqueous solutions (20 wt %) was performed from 10 to 60 °C at a heating rate of 5 °C/min in a nitrogen atmosphere. The heat of fusion per gram of MP-Cl, MP-N_3_ and MP-NH_2_ was calculated according to the area under the peak. The crystallinity of MP-Cl, MP-N_3_ and MP-NH_2_ in aqueous solutions (20 wt %) was measured by X-ray diffraction (XRD; D/MAX-2500V/PC, Rigaku, Tokyo, Japan). A Ni filter at 35 kV and 15 mA generated the radiation. The samples were placed in a quartz sample holder and scanned from 10 to 60° at a scanning rate of 5°/min. The electrophoretic mobility of 0.1 wt % MP-Cl, MP-N3 and MP-NH2 copolymer solutions was measured by electrophoretic light scattering photometer (ELSZ-1000; Otsuka Electronics, Osaka, Japan) at 37 °C. The measured mobility was converted to zeta potential of copolymer solutions.

### 2.3. Synthesis of MPEG-b-PCL Diblock Copolymer (MP)

MP diblock copolymers with PCL molecular weights (2410 g/mol) using MPEG (750 g/mol) were prepared via a previously reported block copolymerization method [8].

### 2.4. Preparation of 2-Chloro-ε-caprolactone (fCL)

All glasses were heated in a vacuum and flushed with dry nitrogen stream for drying. All reactions were conducted in dry nitrogen stream. 3-Chloroperoxybenzoic acid (5.2 g, 30 mmol) was placed in a 100 mL flask and dissolved at CH_2_Cl_2_ (20 mL). 2-Chlorocyclohexanone (2 g, 15 mmol) was added to the solution, which was maintained at room temperature. After 48 h, the resulting solution was washed with a saturated aqueous NaHSO_3_ solution and an aqueous NaHCO_3_ solution several times, followed by washing with a brine solution. The organic phase was dried over anhydrous magnesium sulfate and concentrated by evaporation, yielding fCL, i.e., a light yellowish viscous liquid. The obtained mixture was purified by silica-gel column chromatography using a solution of *n*-hexane and ethyl acetate (*v*/*v* = 70/30, *R*_f_ = 0.6) as an eluent, yielding 1.07 g (7.2 mmol, 48%) of clear liquid. ^1^H-NMR (CDCl_3_): 4.81 (*s*, 1H, –C*H*(Cl)–) 4.61 (*t*, 2H, –OC*H*_2_–), 4.22 (*t*, 2H, –OC*H*_2_–), 2.05 (*m*, 4H, –C*H*_2_–, –C*H*_2_–), 1.82 (*m*, 4H, –C*H*_2_–). ^13^C-NMR (CDCl_3_): 169.6, 69.2, 58.8, 34.0, 29.1, 26.1. Anal. Calcd. for C_6_H_9_ClO_2_, C: 48.50, H: 6.11. Found: C: 48.27, H: 6.02.

### 2.5. Synthesis of MPEG-b-(poly(ε-caprolactone)-ran-poly(2-chloride-ε-caprolactone)) (MPEG-b-(PCL-ran-PfCL)) (MP-Cl) Diblock Copolymers

All glasses were heated in a vacuum and dried via flushing with dry nitrogen stream. We used the typical polymerization process for producing MP-Cl-97/3 with a CL/fCL ratio of 97/3 using MPEG (750 g/mol) as an initiator, as follows. MPEG (1.6 g, 2.1 mmol) and toluene (55 mL) were added into a flask. Azeotropic distillation was performed to remove water from the MPEG and toluene. Then toluene was removed via distillation. CL (4.92 g, 43 mmol) and fCL (0.2 g, 1.3 mmol) were introduced to the MPEG at room temperature under dry nitrogen stream. The polymerization was started by the addition of a 1.0 M solution of HCl·Et_2_O (3.2 mL, 3.2 mmol) at room temperature. After 24 h, the mixture was poured into a mixture of *n*-hexane to precipitate a polymer. The precipitated polymers were obtained from the supernatant by decantation, dissolved in CH_2_Cl_2_, and then filtered. The resulting polymer solution was concentrated by rotary evaporation and dried in a vacuum, yielding a colorless copolymer. In the same way, MP-Cl copolymers (MP-Cl-97/3, MP-Cl-95/5, MP-Cl-90/10 and MP-Cl-85/15) were prepared. The molecular weights and ratios of the PCL and PCL-Cl segments in the copolymers were determined by comparing the intensity of the carbonyl carbons in PCL and PCL-Cl segments at δ = 172.7 and 169.6 ppm, respectively, at ^13^C-NMR peaks.

### 2.6. Synthesis of MPEG-b-(poly(ε-caprolactone)-ran-poly(2-azide-ε-caprolactone)) (MPEG-b-(PCL-ran-PCL-N_3_)) (MP-N_3_) Diblock Copolymers

MP-Cl (5 g, 1.6 mmol, with 0.05 mmol concentration of Cl group) and DMF (23 mL) were inserted into a flask. Sodium azide (0.01 g, 0.1 mmol) was added to the MP-Cl solution at room temperature under nitrogen, and the mixture was stirred at 65 °C for 48 h. The reaction mixture was then poured into a mixture of *n*-hexane and ethyl ether (*v*/*v* = 4/1) to precipitate the polymer, which was separated from the supernatant by decantation. The obtained polymer was redissolved in CH_2_Cl_2_. The resulting solution was washed with a brine solution. The organic phase was concentrated using a rotary evaporator and dried in vacuum to obtain a yellowish copolymer.

### 2.7. Synthesis of MPEG-b-(poly(ε-caprolactone)-ran-poly(2-amine-ε-caprolactone)) (MPEG-b-(PCL-ran-PCL-NH_2_)) (MP-NH_2_) Diblock Copolymers

MP-N_3_ (4 g, 1.3 mmol, concentration of azide group: 0.04 mmol) and DMF (20 mL) were introduced into a flask. Triphenylphosphine (0.02 g, 0.08 mmol) was added to the MP-N_3_ solution at room temperature under nitrogen, and the mixture was stirred at room temperature. After 10 h, H_2_O (1 mL) was poured into the reaction mixture, and the mixture was stirred for 1 h. The reaction mixture was then poured into a mixture of *n*-hexane and ethyl ether (*v*/*v* = 4/1) to precipitate the polymer, which was separated from the supernatant by decantation. The obtained polymer was redissolved in CH_2_Cl_2_. The resulting solution was washed with a saturated aqueous NaHCO_3_ solution several times. The organic phase was dried over anhydrous magnesium sulfate and concentrated by evaporation to obtain an opaque copolymer.

### 2.8. Determination of Sol-to-Gel Phase-Transition Times via Tilting Experiment

To examine the gelation time of the MP-Cl, MP-N_3_ and MP-NH_2_ copolymer solutions, the copolymers were dissolved at 20 wt % in 4 mL vials at 80 °C in deionized water (DW). The MP-Cl, MP-N_3_ and MP-NH_2_ copolymers were obtained as homogeneous opaque emulsions and were stored at 4 °C. After 48 h, the homogeneous opaque emulsions in the vials were gently stirred at room temperature, and the vials were immediately immersed in a 37 °C water bath. While the vials were maintained at 37 °C, the time taken by the emulsions to stop exhibiting flow was determined and defined as the gelation time.

### 2.9. Determination of Sol-to-Gel Phase-Transition Times via Rheological Measurement

The gelation time of the MP-Cl, MP-N_3_ and MP-NH_2_ copolymers was measured using a rheometer (MCR 102, Anton Paar, Ostfildern, Germany) with peltier temperature-control system for bottom plates and a 25.0 mm stainless-steel parallel-plate measuring system. The storage modulus G′ and loss modulus G″ were measured under a 1.0% strain level at 37 °C and calculated using the software of the instrument. The gelation time was determined at the crossover point of the G′ and G″ curves.

### 2.10. Viscosity Measurements

MP-Cl, MP-N_3_, and MP-NH_2_ copolymers (0.5 g, 0.15 mmol) was dissolved in a 4 mL vial at 80 °C to obtain a concentration of 20 wt % by using DW and then stored at 4 °C. After 48 h, the viscosities of the copolymer solutions were measured using a Brookfield Viscometer DV-III Ultra, which was equipped with a programmable rheometer and circulating baths featuring a programmable controller (TC-502P). The viscosity of the MP-Cl, MP-N_3_ and MP-NH_2_ copolymer solutions was determined using a T-F spindle at 0.1 rpm from 10 to 60 °C in increments of 1 °C.

## 3. Results and Discussion

### 3.1. Preparation and Characterization of MP-Cl, MP-N_3_, and MP-NH_2_

Firstly, MP-Cl was synthesized at room temperature via the ring-opening polymerization of the monomer CL and fCL using the terminal alcohol of MPEG as the initiator in the presence of HCl·Et_2_O. The colorless MP-Cl diblock copolymers were obtained in almost quantitative yield.

**Figure 2:**
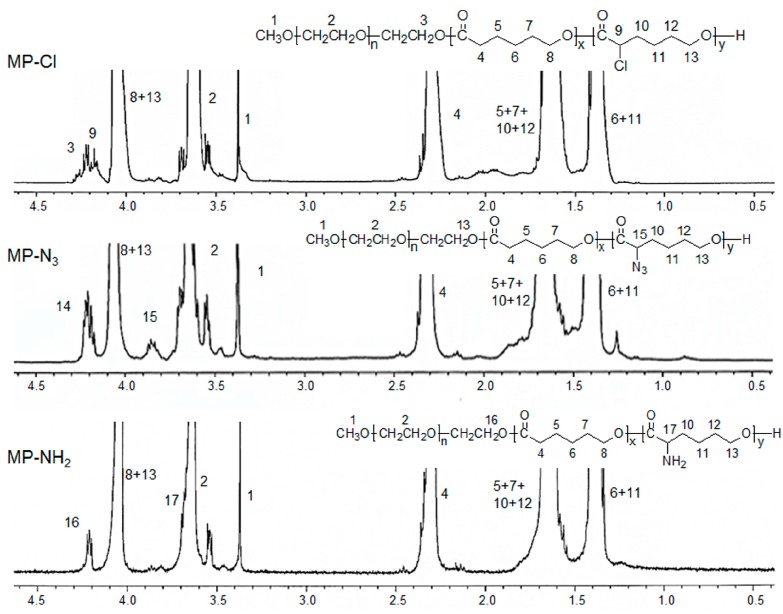
^1^H-NMR spectra of MP-Cl, MP-N_3_ and MP-NH_2_ diblock copolymers.

**Figure 3:**
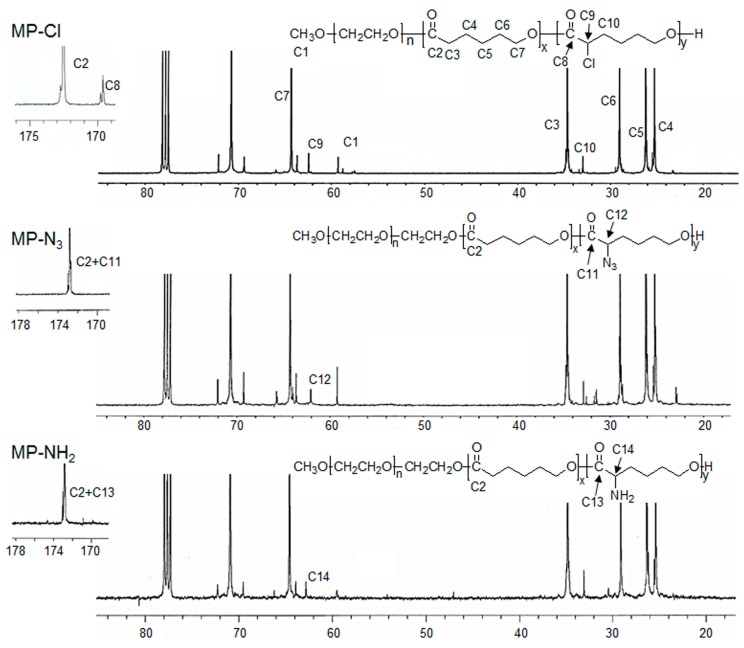
^13^C-NMR spectra of MP-Cl, MP-N_3_ and MP-NH_2_ diblock copolymers.

^1^H- and ^13^C-NMR of the MP-Cl diblock copolymers exhibited characteristic peaks of MPEG, PCL, and PCL-Cl (Figure 2 and Figure 3). The methoxy, methylene, and methine protons 1, 3 and 9 were observed at δ = 3.48, 4.21, and 4.18 ppm at ^1^H-NMR peaks. The carbonyl carbons (–C(=O)) of PCL and PCL-Cl segments were observed at δ = 172.7 and 169.6 ppm at ^13^C-NMR peaks, respectively. ^13^C-NMR of the MP-Cl diblock copolymer also showed a peak that could be assigned to –C(H)(Cl)– at δ = 62.15 ppm. The ratios of the PCL and PCL-Cl segments in the copolymers were determined according to the carbon-integration ratios of carbonyl in the PCL and PCL-Cl segments, which agreed well with the expected values. Additionally, the molecular weights of MP-Cl determined by NMR spectroscopy were close to theoretical values calculated from different ratios of CL and fCL. This indicates that the polymerization procedure yielded targeted MP-Cl diblock copolymers with a PCL-Cl content of 3–15 mol % in the PCL segment. The synthesis results for MP-Cl with Cl pendant group contents of 3–15 mol % in the PCL segment was summarized in Supplementary Materials Table S1. This result clearly showed that we successfully prepared the MP-Cl by metal-free ring-opening polymerization of the monomer CL and fCL.

Next, MP-N_3_ was obtained as light yellowish diblock copolymers through the modification of MP-Cl using sodium azide. The synthesis results for MP-N_3_ with azide pendant group contents of 3–15 mol % in the PCL segment was summarized in Supplementary Materials Table S1. ^1^H- and ^13^C-NMR of the MP-N_3_ diblock copolymer also exhibited characteristic peaks corresponding to PCL, MPEG, and MP-N_3_ (Figure 2 and Figure 3). Specially, the methine proton 15 of the –C(H)(N_3_)– was observed at δ = 3.85 ppm. The MP-N_3_ diblock copolymer showed a new ^13^C-NMR peak owing to –C(H)(N_3_)– at δ = 61.82 ppm but complete disappearance of the signal at δ = 169.6 ppm, assigned to –C(=O) of the PCL-Cl segments. The Fourier transform infrared (FT-IR) spectra of the MP-N_3_ diblock copolymer exhibited characteristic peaks around 2105 cm^−1^, which are assigned to the stretching of the azide group (Supplementary Materials Figure S1). This indicates that the chloride group on MP-Cl was quantitatively changed into the azide group.

Finally, an MP-NH_2_ diblock copolymer was obtained by the subsequent modification of MP-N_3_ using the Staudinger reaction. The MP-NH_2_ diblock copolymer had the appearance of –C(H)(NH_2_) at δ = 62.97 ppm of ^13^C-NMR. In addition, the azide group was completely disappeared in the FT-IR spectra of the MP-NH_2_ diblock copolymer.

The obtained MP-N_3_ and MP-NH_2_ diblock copolymers with different composition ratios were compared with a corresponding diblock copolymer via elemental analysis (Supplementary Materials Table S2). The elemental analysis of the MP-N_3_ and MP-NH_2_ diblock copolymers showed values almost similar to the expected values at all composition ratios. This result indicates that the pendant azide and amine groups were stoichiometrically modified into MP diblock copolymers and demonstrates that the procedure described here generated the azide and amine-modified MP diblock copolymers.

**Figure 4:**
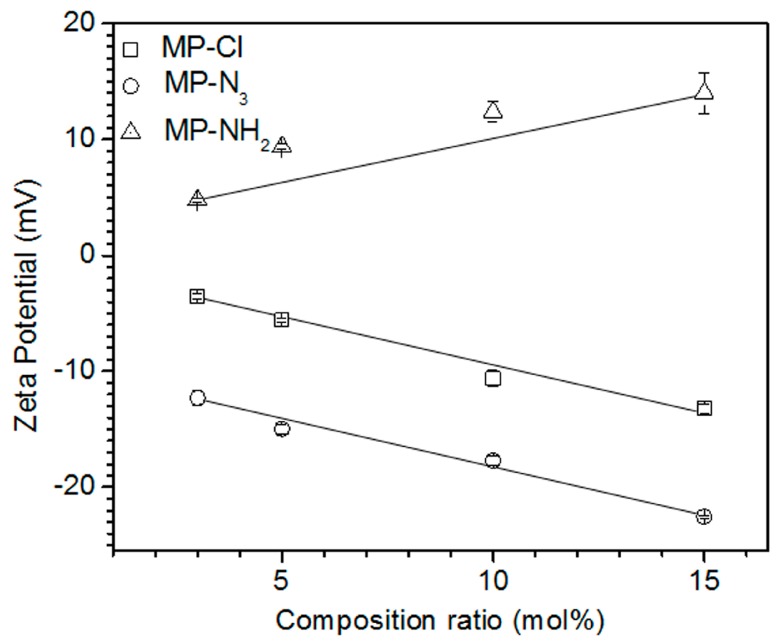
Zeta potential versus block composition ratios for MP-Cl, MP-N_3_ and MP-NH_2_ diblock copolymers with 3–15 mol % of chloride, azide and amine pendant groups.

To examine the electrostatic properties according to the variation in the composition ratios of chloride, azide, and amine pendant groups, the zeta potential was measured in solutions of MP, MP-Cl, MP-N_3_ and MP-NH_2_ diblock copolymers (Figure 4). The zeta potential of the MP solution was −2.7 mV. The MP-Cl and MP-N_3_ exhibited negative zeta potentials at all composition ratios because of the negative properties of the chloride and azide groups on MP-Cl and MP-N_3_. The negative zeta potentials of the chloride- and azide-group on alkyl or polymer chain were reported [20,21,22,23]. The zeta potentials of MP-N_3_ were lower than those of MP-Cl at the same composition ratios. The zeta potentials of the MP-Cl and MP-N_3_ solutions decreased almost linearly from −3.6 to −13.2 mV and −12.3 to −22.5 mV, respectively, for the chloride- and azide-group contents of 3–15 mol %. MP-NH_2_ had a positive zeta potential of 4.8 mV and increased almost linearly to 14 mV as the amine content increased.

These results indicate that the electrostatic properties of the MP-Cl, MP-N_3_ and MP-NH_2_ copolymers were affected by the variation of the chloride, azide and amine pendant groups, as well as the amount of pendant groups in the structure.

### 3.2. Solution Properties of MP-Cl, MP-N_3_ and MP-NH_2_

**Figure 5:**
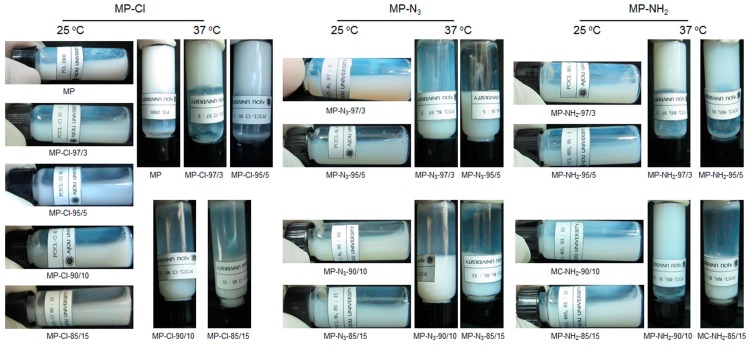
Images of MP-Cl, MP-N_3_, and MP-NH_2_ diblock copolymer solutions with 3–15 mol % chloride, azide and amine pendant groups at 25 and 37 °C.

Aqueous solutions of the MP-Cl, MP-N_3_ and MP-NH_2_ diblock copolymers were prepared by dissolving them in DW at 80 °C. The aqueous MP-Cl, MP-N_3_ and MP-NH_2_ solutions formed opaque emulsions at room temperature (Figure 5). The solution properties of all the copolymers were determined according to the emulsion formation time, the phase transition, and the gelation time (Supplementary Materials Table S3). The MP has an emulsion formation time of 20 min and a gelation time below 10 s.

Then, MP, MP-Cl, MP-N_3_ and MP-NH_2_ diblock copolymers in the emulsion-sol state were individually incubated at 37 °C. The time taken by incubation at 37 °C to stop exhibiting a flow of the emulsions was determined as the gelation time, as summarized in Supplementary Materials Table S3.

The emulsion formation time of MP-Cl decreased as the chloride-group content increased. The emulsion formation time of MP-Cl with a chloride group content of 3 mol % was ~15 min, and the time sharply decreased to 3 min as the chloride group content increased. The phase transition of MP-Cl with chloride group contents of 3 and 5 mol % was observed at 37 °C, and the gelation time was ~30 s. MP-Cl with chloride group contents of 10 and 15 mol % had a short emulsion formation time and no phase transition, probably owing to easy formation of emulsion of the MP-Cl diblock copolymers in the aqueous solution.

The emulsion formation time of MP-N_3_ decreased as the azide group content increased. The emulsion formation of MP-N_3_ was less than 10 min. In addition, for MP-N_3_, at all azide group contents, there was no phase transition, owing to easy formation of emulsion of MP-N_3_ diblock copolymers in the aqueous solution.

MP-NH_2_ with amine group contents of 3 and 5 mol % had solubilization times of 10 and 5 min, respectively, and a solution-to-hydrogel phase transition at 37 °C. The gelation time increased from 20 to 50 s as the amine group content increased. MP-NH_2_ with an amine group content of 15 mol % formed emulsion in the aqueous solution and thus showed no phase transition.

These results indicate that the phase transition and the gelation time of the MP-Cl, MP-N_3_ and MP-NH_2_ copolymers were affected by the variation of the chloride, azide and amine pendant groups as well as the amount of pendant groups present in their structure.

### 3.3. Rheological Properties of MP-Cl, MP-N_3_ and MP-NH_2_

**Figure 6:**
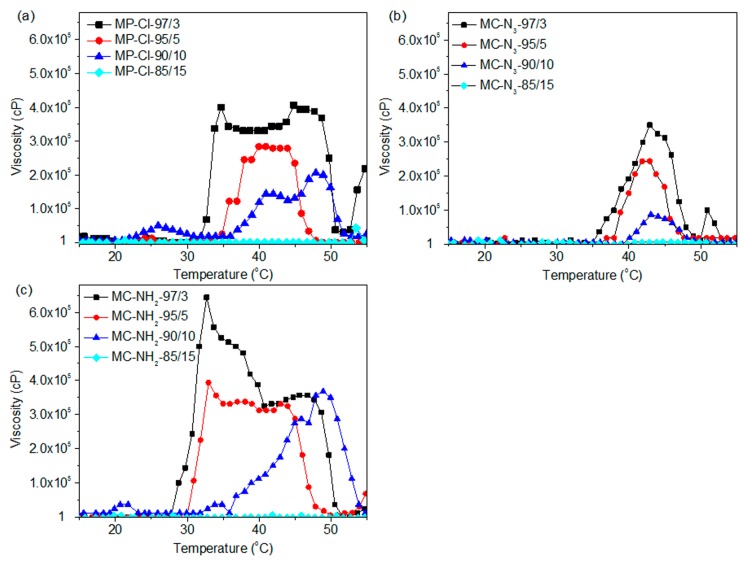
Viscosity-versus-temperature curves of (**a**) MP-Cl with 3–15 mol % of chloride pendant groups, (**b**) MP-N_3_ with 3–15 mol % of azide pendant groups, and (**c**) MP-NH_2_ with 3–15 mol % of amine pendant groups.

The viscosities of a series of MP-Cl, MP-N_3_ and MP-NH_2_ copolymers containing various pendant groups were measured as a function of temperature. Figure 6 shows the viscosity-temperature curves of MP-Cl, MP-N_3_ and MP-NH_2_ with 3–15 mol % of chloride, azide and amine pendant groups. Below 28 °C, the viscosities of the MP-Cl, MP-N_3_ and MP-NH_2_ solutions at all pendant group ratios were in the range of 1–1000 cP, demonstrating that this fluid-like zone corresponded to an emulsion of MP-Cl, MP-N_3_ and MP-NH_2_.

At 28–50 °C, the viscosities of the MP-Cl, MP-N_3_ and MP-NH_2_ solutions increased, indicating that in this zone, the copolymers became gel-like and that the interactions between the hydrophobic blocks in MP-Cl, MP-N_3_ and MP-NH_2_ were strengthened in this temperature range. As the content of pendant groups increased, the onset temperatures increased, and maximum viscosities decreased.

**Figure 7:**
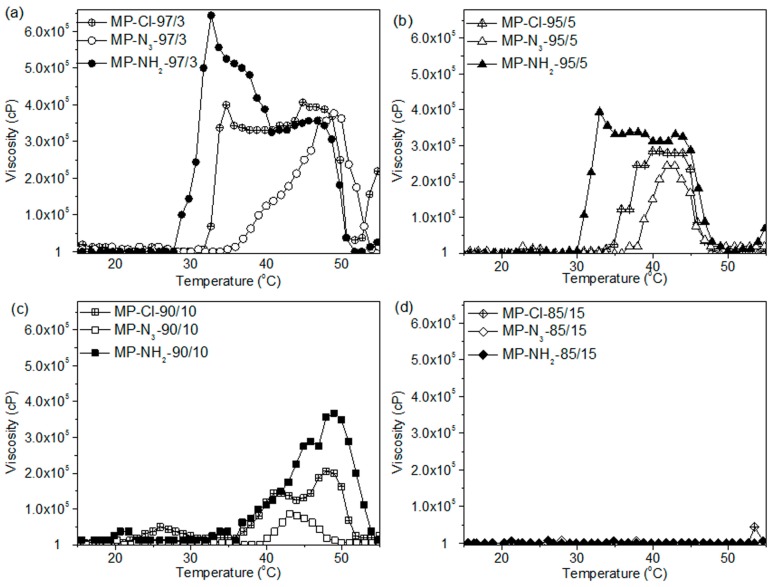
Viscosity-versus-temperature curves of MP-Cl, MP-N_3_, and MP-NH_2_ diblock copolymer solutions with (**a**) 3, (**b**) 5, (**c**) 10, and (**d**) 15 mol % of pendant groups.

For MP-Cl copolymers (Figure 7a), the onset temperatures of gelation increased as the amount of chloride pendant groups increased in the MP-Cl copolymer, except in the case of MP-Cl with 15 mol % of chloride. MP-Cl with 3, 5 and 10 mol % of chloride exhibited onset temperatures of 33, 35 and 38 °C, respectively. The order of maximum viscosities of MP-Cl at 37 °C was 3 > 5 > 10 mol % of the chloride group in the MP-Cl copolymer. MP-Cl with 15 mol % of the chloride group was dissolved in water, yielding a viscosity of 1 cP, and no phase-transition behavior was observed.

MP-N_3_ and MP-NH_2_ exhibited similar onset temperatures and orders of viscosity at 37 °C to MP-Cl copolymers (Figure 7b,c). A solution-to-hydrogel phase transition was observed upon introduction of chloride, azide and amine up to concentrations of 10 mol %. MP-Cl, MP-N_3_ and MP-NH_2_ with 15 mol % of chloride, azide and amine showed a viscosity of 1 cP, indicating no phase transition.

The copolymers in order of increasing onset temperatures was MP-NH_2_ < MP-Cl < MP-N_3_ up to a concentration of 10 mol %. MP-Cl, MP-N_3_ and MP-NH_2_ copolymers with 15 mol % of pendant groups exhibited a viscosity of 1 cP, indicating no phase transition. The maximum viscosities decreased in the order of MP-NH_2_ > MP-Cl > MP-N_3_.

The order of the onset temperatures and maximum viscosities were similar to the order of the zeta potentials for the MP-Cl, MP-N_3_ and MP-NH_2_ copolymers. For MP-NH_2_ copolymers, a cationic pendant amine group on the PCL can stabilize aggregation between amine groups on the hydrophobic PCL segments via hydrogen bonding, but higher concentrations of amine pendant groups in aqueous solutions induce plenty of hydrogen bonding with surrounding large water.

A negative pendant chloride group on the PCL slightly stabilized the aggregation between the hydrophobic PCL segments at low chloride contents but induced the destabilization of aggregation between PCL segments at high chloride contents. Additionally, the large negative potential of the azide group on the PCL showed less stabilization than the chloride group at all pendant contents.

Collectively, the pendant chloride, azide and amine groups of the MP-Cl, MP-N_3_ and MP-NH_2_ copolymers altered the packing or aggregation in the diblock copolymer solution, which was mediated by intra- and inter-molecular hydrophobic interactions or hydrogen bonding between pendant groups. These results suggest that pendant groups or the compositional ratio of MP-Cl, MP-N_3_ and MP-NH_2_ can be used to adjust the temperature-responsive window.

### 3.4. Crystallization of MP-Cl, MP-N_3_ and MP-NH_2_

As described in the previous section, the phase transition of the MP-Cl, MP-N_3_ and MP-NH_2_ copolymers was altered by pendant groups through intra- and inter-molecular aggregation of the PCL blocks. To understand the intra- and inter-molecular aggregation between the hydrophobic domains of MP-Cl, MP-N_3_ and MP-NH_2_, the crystalline properties of MP-Cl, MP-N_3_ and MP-NH_2_ with 3–15 mol % of chloride, azide and amine pendant groups were examined according to the thermal properties via DSC and XRD in the bulk and aqueous solutions.

The thermal properties of the copolymers were measured as summarized in Supplementary Materials Table S4. The DSC thermograms of the diblock copolymer showed the peak assignable to melting of the hydrophobic domains, which could be attributed to the crystallization enthalpy of the hydrophobic domains. The thermal analysis indicated that the MP diblock copolymer exhibited a melting temperature of 56 °C and that the crystallization enthalpy of the MP diblock copolymers was 70 J/g. The solutions of the MP diblock copolymer exhibited a crystallization enthalpy of 11 J/g at a melting temperature of 47.6 °C. The degrees of crystallinity in the aqueous-solution copolymers were estimated to be 37% via XRD.

MP-Cl diblock copolymers with 3–15 mol % of chloride pendant groups on the PCL segment showed a decreasing crystallization enthalpy—from 53 to 18 J/g—with increasing number of chloride pendant groups. The solutions of MP-Cl diblock copolymers also showed a decrease of the crystallization enthalpy, which reached zero at a chloride group content of 15 mol %, probably because of the complete phase mixing of the MP-Cl diblock copolymers in the aqueous solution.

The degrees of crystallinity in the aqueous-solution state of the MP-Cl diblock copolymers decreased from 32% to 17% with increasing chloride pendant groups. These results indicate that the chloride group on MP prohibited aggregation between the hydrophobic PCL segments. In the bulk and aqueous phases, MP-N_3_ with 3–15 mol % of azide groups in the PCL segment showed almost the same behavior as the crystallization enthalpy and the degrees of crystallinity of the MP-Cl diblock copolymers. This result indicates that the azide group in the PCL segment prohibited aggregation between the hydrophobic segments.

The thermal properties of MP-NH_2_ copolymers containing 3, 5 and 10 mol % of amine groups showed increases in the crystallization enthalpy—from 52 to 77 J/g—and the degrees of crystallinity—from 31% to 36%—with increasing amine pendant groups, but with 15 mol % of amine groups, the crystallization enthalpy decreased to 33 J/g, and the degree of crystallinity decreased to 22%.

According to the XRD analysis of the MP-Cl diblock copolymer, the total crystallinity decreased from 32% to 17% as the amount of the chloride pendant groups increased. For the MP-N_3_ diblock copolymer, the total crystallinity decreased from 30% to 23% as the amount of the azide pendant groups increased.

For the MP-NH_2_ diblock copolymer, the total crystallinity increased from 31% to 36% as the amount of the amine group increased, except in the case of MP-NH_2_-85/15. DSC and XRD indicate that the crystallinity increased with the amount of amine pendant groups. This indicates that the crystallinity of the MP-Cl, MP-N_3_ and MP-NH_2_ copolymers was affected by the variation of the chloride, azide and amine pendant groups, as well as the amount of pendant groups in their structure.

The plausible mechanism for thermogelling of the amphiphilic diblock copolymers is proposed by the formation of the microphase-separated micelles and the interconnection between micelles [24]. Thus, large crystallinity of hydrophobic domains can enhance and stabilize packing of interconnected micelles. The presence of crystallinity in aqueous medium in this work strongly supports the idea that the MP-Cl, MP-N_3_ and MP-NH_2_ diblock copolymer solutions stabilize the aggregation of hydrophobic domains via intra- and inter-molecular interactions. However, the crystallinity of hydrophobic domains of the MP-Cl, MP-N_3_ and MP-NH_2_ diblock copolymer solutions could rise or decline because the functional pendant groups on MP diblock copolymers could stabilize or destabilize diblock copolymer chain aggregation.

Collectively, the pendant chloride and azide groups induced weakened and/or prohibited aggregation between the hydrophobic segments. The amine groups enhanced aggregation between the hydrophobic segments via intra- and inter-molecular hydrogen-bonding interactions but weakened the aggregation between the hydrophobic segments via complete phase mixing at an amine-group content of 15 mol %.

## 4. Conclusions

We successfully prepared the MP-Cl by metal-free ring-opening polymerization of the monomer CL and fCL. Then, MP-N_3_ and MP-NH_2_ with azide and amine pendant group contents of 3–15 mol % in the PCL segment showed a solution-to-hydrogel phase transition that depended on both the type of pendant groups and their content. The solution-to-hydrogel phase transition temperature was adjusted by tailoring the crystallinity and hydrophobicity of the copolymers in aqueous solutions. Thus, we consider that the proposed MP-Cl, MP-N_3_ and MP-NH_2_ copolymers with various pendant groups can be utilized as injectable drug and cell carriers. Meanwhile, further experiments will be needed to investigate the pH-responsible thermogelling behavior and their solution properties of pendant group-functionalized diblock copolymers.

## Supplementary Materials

The following are available online at www.mdpi.com/2073-4360/9/6/239/s1, Figure S1: FT-IR spectra of MP-Cl, MP-N_3_, and MP-NH_2_ diblock copolymers, Table S1: Synthesis of MP-Cl, MP-N_3_, and MP-NH_2_ diblock copolymers, Table S2: Elementary analysis of MP-N3 and MP-NH2 diblock copolymers, Table S3: Thermogelling properties of MP-Cl, MP-N_3_, and MP-NH_2_ diblock copolymers, Table S4: Thermal properties of MP-Cl, MP-N3, and MP-NH2 diblock copolymers.
