# Supplementary material for: Preparation of Pendant Group-Functionalized Diblock Copolymers with Adjustable Thermogelling Behavior
Source: Polymers (Basel). 2017 Jun 20;9(6):239. doi: 10.3390/polym9060239 (PMC6432020; doi:10.3390/polym9060239)
Supplement: Supplementary file 1 [file polymers-09-00239-s001.pdf]

Supplementary Material

# Preparation of pendant group-functionalized diblock copolymers with adjustable thermogelling behavior

**Bo Keun Lee, Ji Hoon Park, Seung Hun Park, Jae Ho Kim, Se Heang Oh, Sang Jin Lee, Bun Yeoul Lee, Moon Suk Kim***

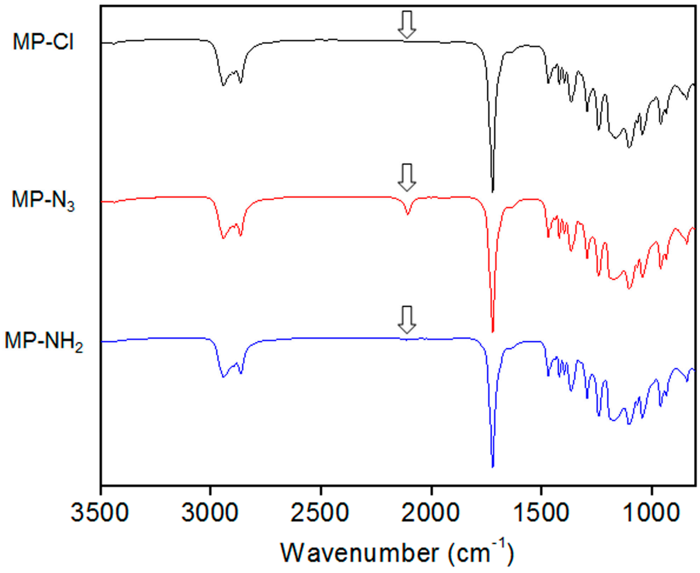


**Figure S1.** FT-IR spectra of MP-Cl, MP-$N_3$, and MP-$NH_2$ diblock copolymers.

**Table S1.** Synthesis of MP-Cl, MP-$N_3$, and MP-$NH_2$ diblock copolymers.

| No | [CL]/[fCL] molar ratio | $M_{n, calcd}$ MPEG-polyester (g/mol) | $M_{n, NMR}$ [a] MPEG-polyester (g/mol) | Yield (%) | $M_w/M_n$ [b] |
|---|---|---|---|---|---|
| MP | 100/0 | 750-2400 | 750-2410 | 96 | 1.2 |
| MP–Cl–97/3 | 97/3 | 750-2410 | 750-2380 | 91 | 1.4 |
| MP–$N_3$–97/3 | | 750-2420 | 750-2410 | 81 | 1.4 |
| MP–$N_3$–97/3 | | 750-2380 | 750-2400 | 72 | 1.8 |
| MP–Cl–95/5 | 95/5 | 750-2430 | 750-2540 | 94 | 1.4 |
| MP–$N_3$–95/5 | | 750-2570 | 750-2550 | 87 | 1.6 |
| MP–$N_3$–95/5 | | 750-2540 | 750-2540 | 68 | 1.7 |
| MP–Cl–90/10 | 90/10 | 750-2480 | 750-2610 | 94 | 1.3 |
| MP–$N_3$–90/10 | | 750-2660 | 750-2630 | 84 | 1.3 |
| MP–$N_3$–90/10 | | 750-2600 | 750-2670 | 74 | 1.5 |
| MP–Cl–85/15 | 85/15 | 750-2420 | 750-2430 | 96 | 1.3 |
| MP–$N_3$–85/15 | | 750-2515 | 750-2520 | 89 | 1.3 |
| MP–$N_3$–85/15 | | 750-2440 | 750-2440 | 80 | 1.6 |

[a] Determined from $^1$H NMR spectra.

[b] Measured by means of gel-permeation chromatography (based on standard polystyrene).

**Table S2.** Elementary analysis of MP-N3 and MP-NH2 diblock copolymers.

| | 97/3 | | 95/5 | | 90/10 | | 85/15 | |
|---|---|---|---|---|---|---|---|---|
| | MP-$N_3$ | MP-$NH_2$ | MP-$N_3$ | MP-$NH_2$ | MP-$N_3$ | MP-$NH_2$ | MP-$N_3$ | MP-$NH_2$ |
| Calc. C | 61.02 | 61.50 | 60.99 | 60.15 | 60.01 | 60.08 | 59.13 | 61.68 |
| Mea. C | 60.96 | 61.28 | 60.66 | 60.72 | 59.96 | 60.05 | 58.73 | 62.47 |
| Calc. H | 8.90 | 8.98 | 8.44 | 8.52 | 8.66 | 8.73 | 8.13 | 8.65 |
| Mea. H | 8.78 | 9.01 | 9.01 | 8.91 | 8.9 | 8.92 | 8.64 | 8.23 |
| Calc. N | 0.61 | 0.21 | 1.07 | 0.35 | 1.42 | 0.47 | 3.47 | 1.2 |
| Mea. N | 0.63 | 0.27 | 0.83 | 0.28 | 1.21 | 0.31 | 3.36 | 0.82 |

**Table S3.** Thermogelling properties of MP-Cl, MP-$N_3$, and MP-$NH_2$ diblock copolymers.

| No | pH [a] | Emulsion formation Time (min) | Phase Transition [b] | Gelation time [c] (s) | Gelation time [d] (s) |
|---|---|---|---|---|---|
| MP | 4.6±0.1 | < 20 | O | ~10 | <1 |
| MP–Cl–97/3 | 5.4±0.1 | < 15 | O | ~30 | <1 |
| MP–$N_3$–97/3 | 6.1±0.02 | < 10 | X | X | X |
| MP–$NH_2$–97/3 | 4.6±0.1 | ~10 | O | ~20 | <1 |
| MP–Cl–95/5 | 4.9±0.1 | ~ 10 | O | ~30 | <1 |
| MP–$N_3$–95/5 | 5.0±0.1 | < 5 | X | X | X |
| MP–$NH_2$–95/5 | 4.2±0.1 | < 5 | O | ~30 | <1 |
| MP–Cl–90/10 | 3.7±0.03 | < 5 | X | X | X |
| MP–$N_3$–90/10 | 3.8±0.1 | < 5 | X | X | X |
| MP–$NH_2$–90/10 | 3.6±0.1 | < 5 | O | ~50 | ~184 |
| MP–Cl–85/15 | 3.5±0.02 | < 3 | X | X | X |
| MP–$N_3$–85/15 | 3.7±0.04 | < 2 | X | X | X |
| MP–$NH_2$–85/15 | 3.4±0.1 | < 3 | X | X | X |

[a] Measured by pH meter (Orion 3-Star, Thermo Scientific, USA).

[b] Solution-to-hydrogel phase transition at 37 °C.

[c] Measured by tilting experiment.

[d] Measured by Anton Paar Viscometer MCR 102.

**Table S4**. Thermal properties of MP-Cl, MP-N3, and MP-NH2 diblock copolymers.

| No | [CL]/[fCL] molar ratio | Bulk state | | | Aqueous solution | | $\chi_c$ [b] |
|---|---|---|---|---|---|---|---|
| | | $T_m$ (°C) | $\Delta H_m$ (J/g) | $T_g$ (°C) | $T_m$ (°C) | $\Delta H_m$ (J/g) | |
| MP | 100/0 | 56.4 | 70.1 | -65.5 | 47.6 | 10.9 | 37 |
| MP–Cl–97/3 | 97/3 | 47.2 | 52.6 | -61.3 | 41.5 | 5.1 | 32 |
| MP–$N_3$–97/3 | | 47.3 | 50.2 | -55.2 | 45.2 | 2.7 | 30 |
| MP–$NH_2$–97/3 | | 48.4 | 51.9 | - | 44.2 | 33.1 | 31 |
| MP–Cl–95/5 | 95/5 | 44.8 | 49.7 | -43.4 | 44.9 | 3.7 | 29 |
| MP–$N_3$–95/5 | | 46.0 | 49.2 | -36.5 | 44.6 | 2.5 | 26 |
| MP–$NH_2$–95/5 | | 48.5 | 67.8 | - | 42.1 | 14.0 | 33 |
| MP–Cl–90/10 | 90/10 | 43.1 | 48.8 | -41.3 | 43.9 | 1.7 | 28 |
| MP–$N_3$–90/10 | | 46.1 | 44.2 | -35.4 | 45.2 | 1.4 | 25 |
| MP–$NH_2$–90/10 | | 52.2 | 76.6 | - | 45.8 | 3.1 | 36 |
| MP–Cl–85/15 | 85/15 | 43.0 | 18.4 | -33.2 | - | - | 17 |
| MP–$N_3$–85/15 | | 45.0 | 33.6 | -18.5 | - | - | 23 |
| MP–$NH_2$–85/15 | | 35.4 | 33.1 | -52.6 | 38.7 | 0.1 | 22 |

[a] Measured by DSC.

[b] $\chi c$ was calculated as the ratio of the crystalline peak areas to the total areas under the scattering curve.
